# Treatment of a femoral shaft fracture in a patient with congenital hip disease: a case report

**DOI:** 10.1186/1752-1947-4-221

**Published:** 2010-07-22

**Authors:** George A Tsakotos, Stefanos D Koutsostathis, George A Macheras

**Affiliations:** 1 4th Orthopaedic Department, KAT Hospital, 2 Nikis str, 145 61 Kifissia, Athens, Greece

## Abstract

**Introduction:**

We present a rare case of two concomitant morbidities treated in one operation. To our knowledge, this is the first report of its kind in the literature.

**Case presentation:**

A 57-year-old Greek woman was admitted to the emergency department having sustained a spiral mid-shaft femoral fracture. She also suffered from an ipsilateral hip congenital dysplasia with ankylosed hip joint due to severe arthritis. She was treated with a total hip arthroplasty using a long stem performing as an intramedullary nail.

**Conclusion:**

We undertook a complex operative treatment of both co-morbidities in a one stage procedure with a satisfactory clinical result.

## Introduction

Femoral shaft fractures are usually high energy traumas, with significant blood loss and pain. These injuries are best treated by closed intramedullary nailing, which stabilizes the fracture site and allows immediate mobilization with full weight bearing. Congenital hip disease is quite common in the adult Greek population. Its incidence has been dramatically reduced as a result of early screening, immediate diagnosis and treatment after birth. Adults with congenital dysplasia usually present with hip arthritis and restrictive pain between the fourth and sixth decade of their life. Total hip arthroplasty in such cases is a demanding and challenging operation.

## Case presentation

A 57-year-old Greek housewife, who was 165 cm tall and weighed 65 kg, was admitted to our hospital after a closed injury of her right femur. She was a married mother with one 18- year-old daughter who was a non-smoker and who did not drink alcohol. She was suffering from an ipsilateral dysplastic hip [1]. As a child she had undergone an unsuccessful operation for a non-defined femoral osteotomy. She had no other significant medical history and received no medication except pain killers. Her right leg was fixed in a flexed and internally rotated deformity. She had been walking with great difficulty for more than 10 years, due to hip and knee stiffness with concomitant severe hip arthritis.

**Figure 1:**
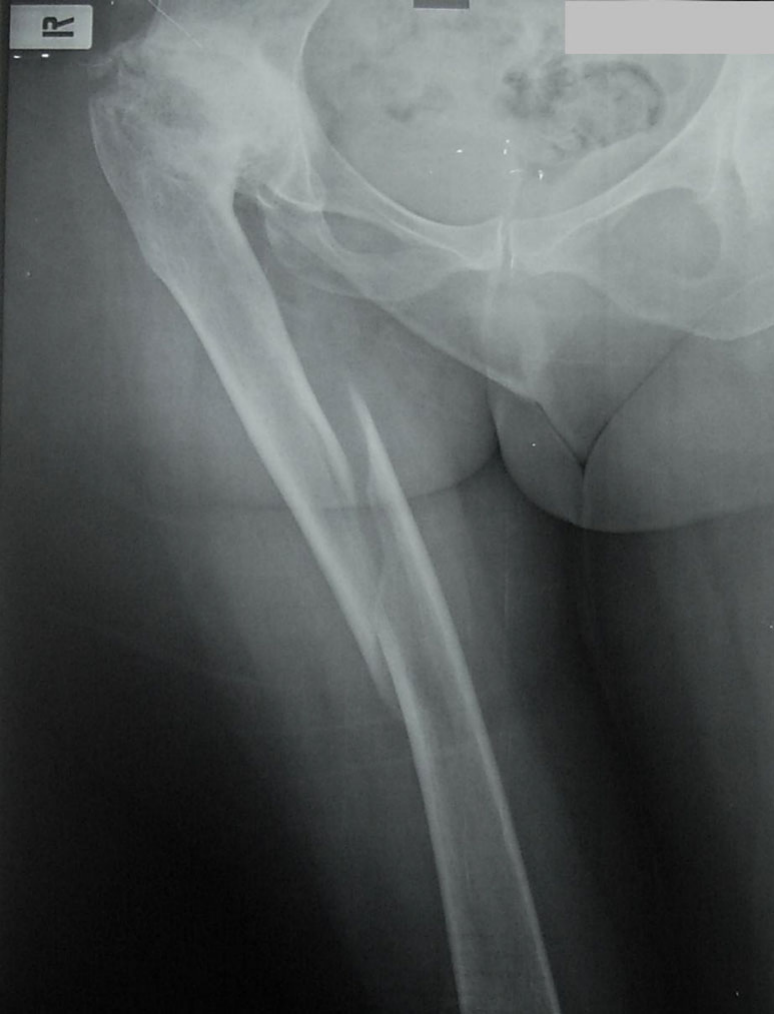
**Initial post traumatic anteroposterior X-ray of the femur**. Hip dysplasia with severe arthritis is recognized. An oblique mid shaft fracture is revealed

She had fallen in her house while walking. On clinical examination, the leg was in fixed flexion with adduction and internal rotation. X-rays revealed an isolated spiral mid-shaft fracture of the right femur (Figure 1): type 0 according to the Winquist-Hansen classification [2] or 32-A1 according to the AO-OTA classification [3].

**Figure 2:**
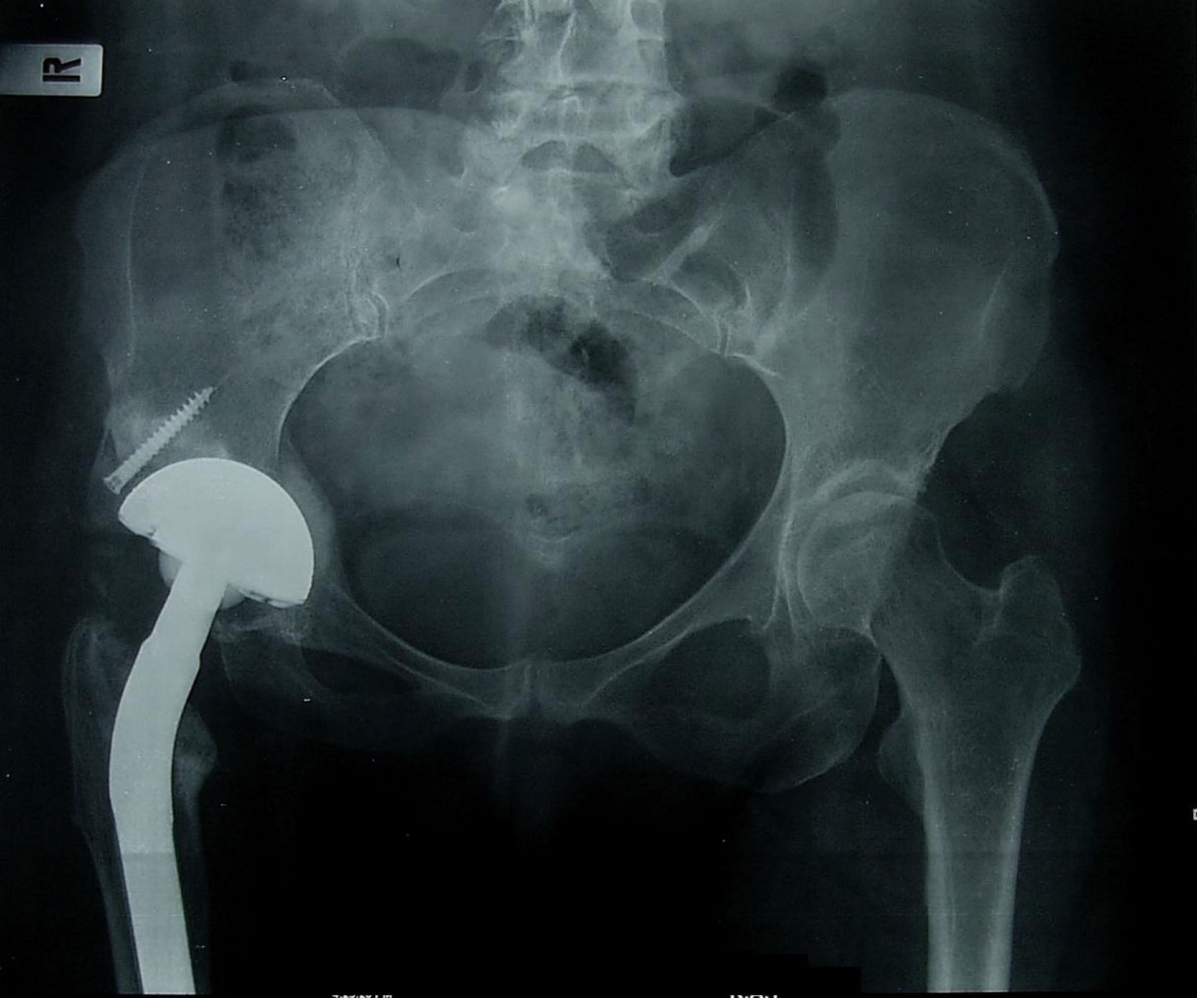
**Pelvis anteroposterior X-ray at 3 months postoperatively**. The cup has no sign of migration. The satisfactory healing process of the morselised graft is seen at the acetabular bed. The structural autograft remains in its initial place held with one screw.

**Figure 3:**
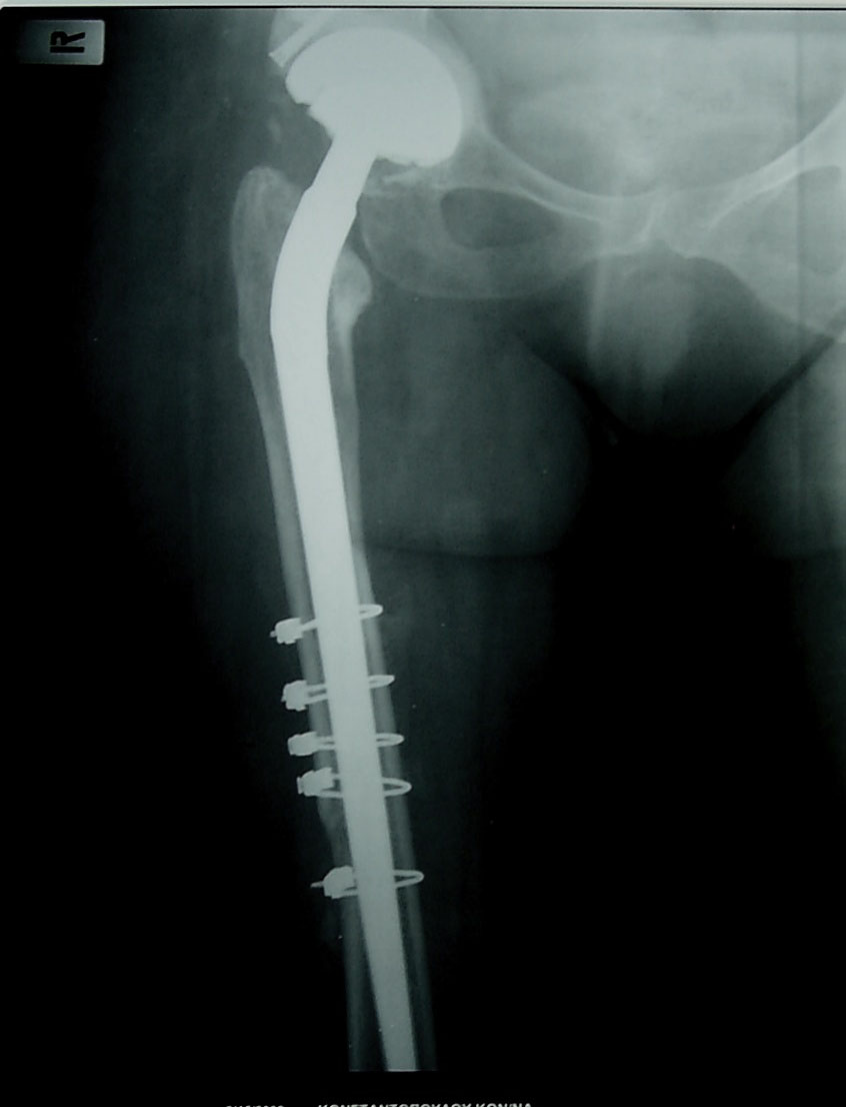
**Anteroposterior X-ray at three postoperative months**. Fracture has healed.

**Figure 4:**
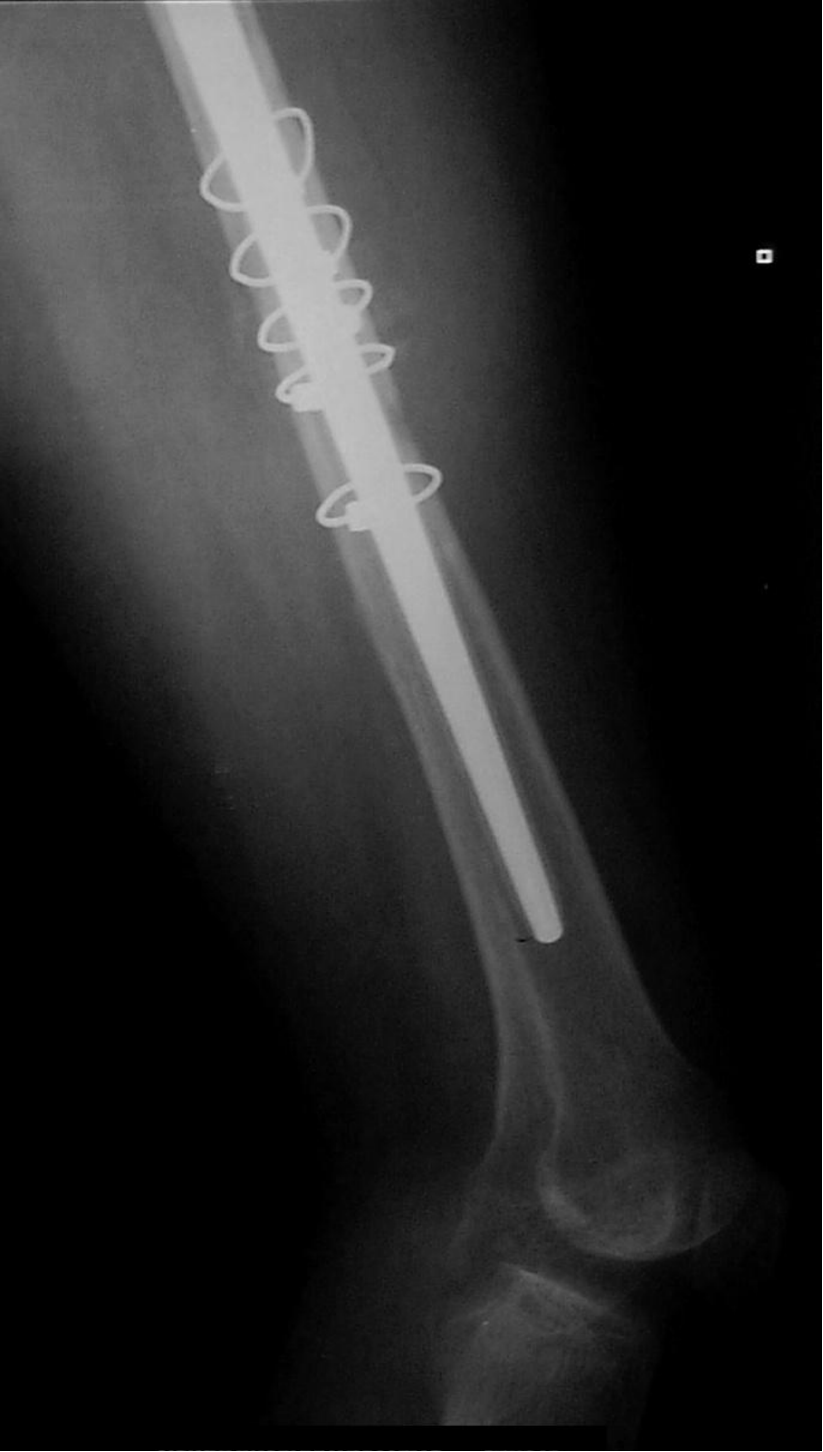
**Lateral X-ray at three postoperative months**. The fracture has healed.

**Figure 5:**
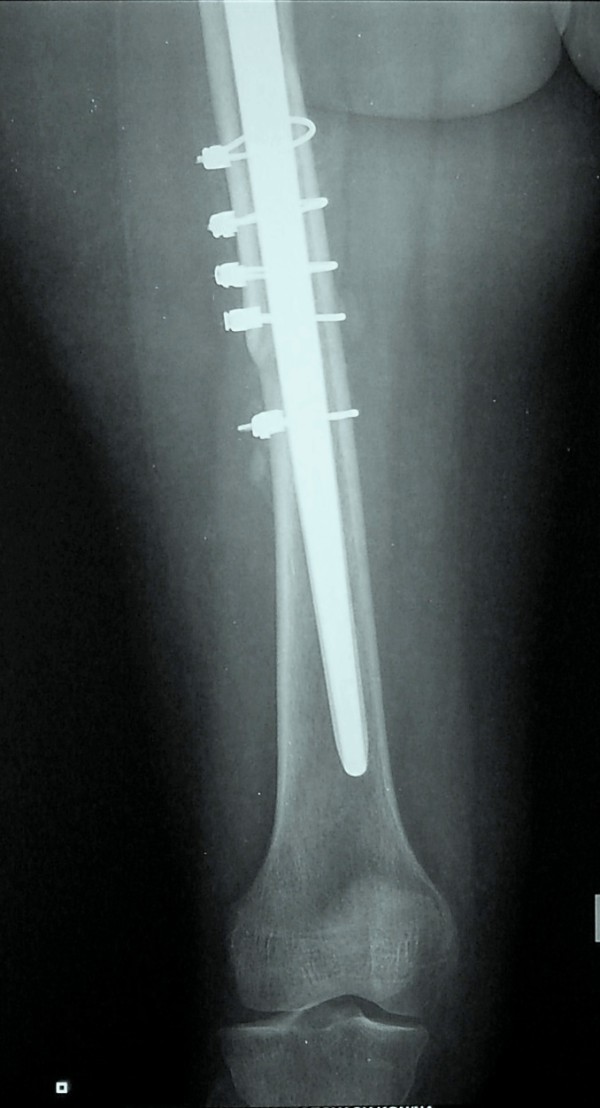
**Distal anteroposterior X-ray at three postoperative months**. The fracture has healed.

We performed a total hip arthroplasty via a posterolateral incision, using a long cementless Wagner stem [4] and a porous tantalum monoblock acetabular cup to address both morbidities. The fixed deformity meant that straight forward hip dislocation was impossible and, therefore, the femoral neck had first to be osteotomised. The cup was placed in the anatomic position. Part of the native head was used as a morselised autograft at the true acetabular bed. The superolateral part of the head was used as a structural graft and secured with one screw. A cup was then inserted in a press fit manner, basing the initial stability on the periphery of the cup. After an additional small incision at the fracture site, the fracture was initially reduced anatomically. Reduction was secured with five cerclage wires and the stem was inserted under direct vision. The operation took 95 minutes. Tissues were sent for culture and histological analysis: the results were negative for tumor or infection, revealing that the fracture was not pathological. The patient received three doses of prophylactic antibiotic and was given low molecular weight heparin for six weeks. There was no leg length discrepancy post-operatively and no complications were recorded. She was mobilized with partial weight bearing the second postoperative day. Full weight bearing was allowed after three weeks, due to the concomitant presence of acetabular graft and diaphyseal cerclage wires. Three months postoperatively, the fracture had healed, the cup showed no signs of migration (Figures 2,3,4,5), there was a normal hip range of motion and patient was walking and free of symptoms.

## Discussion

Femoral shaft fracture is usually caused by a high energy trauma. In this case it is possible that trauma energy was rotational and totally absorbed by the femoral shaft due to the lack of motion at the dysplastic hip, causing a low energy spiral fracture.

There was a debate about the best treatment for this woman. The optimal treatment for femoral mid-shaft fractures is close-locked intramedullary nailing [5]. In this case there was concern about the technical difficulties of antegrade nailing due to the distorted anatomy and the limited ability of intraoperative traction and manipulation because of hip ankylosis in 15° of flexion and as a result of previous surgery. Another option would have been retrograde nailing or a compression plate osteosynthesis. None of the above treatments would have addressed the hip dysplasia and secondary arthritis and stiffness which could have impeded proper weight bearing and lead to the possible mechanical failure of the implants and/or an inability of the fracture to unite. Additionally, it would have been necessary to perform a second operation, even with fracture healing, which would have included material removal and total hip arthroplasty to address the hip dysplasia.

We decided to perform a total hip arthroplasty with a long stem, in order to solve both the patient's problems in one operation. The Wagner stem has been used for many years in revision surgery. We applied a well known technique that has been successful in treating periprosthetic fractures, combining a long stem with cerclage wires. It was essential in this case to use secure open anatomic reduction as it was not a simple femoral fracture which could be treated by a closed intramedullary nailing. The porous tantalum acetabular cup is a very reliable material in dysplastic hip arthroplasties, where acetabular bone stock is poor. It is strongly adherent to bone and, thus, offers excellent initial stability. It is also highly osteoconductive and osteoinductive [6], properties that are important for bone in-growth and long lasting survivorship of the arthroplasty.

## Conclusion

In this case an attempt was made to deal with two different and difficult co-morbidities in one operation. To our knowledge, there has been no similar case reported in the literature. In orthopaedic surgery there is a variety of implants and methods which, used correctly, can help the surgeon to successfully treat high demanding situations.

## Consent

Written informed consent was obtained from the patient for publication of this case report and the accompanying images. A copy of the written consent is available for review by the Editor-in-Chief of this journal.

## Competing interests

The authors declare that they have no competing interests.

## Authors' contributions

GM performed the operation and made the final review. GT analyzed the data and wrote the manuscript. SK performed the follow-up, and reviewed the manuscript. Both GT and SK participated in the operation. All authors have read and approved the final manuscript.
